# Patients’ preferences on atopic dermatitis skincare and social media use: a qualitative study

**DOI:** 10.1186/s12889-025-21640-8

**Published:** 2025-02-05

**Authors:** Roxana Mazilu, Stefanie Ziehfreund, Stephan Traidl, Alexander Zink

**Affiliations:** 1 Department of Dermatology and Allergy, School of Medicine, Technical University of Munich, Biedersteiner Str. 29, 80802 Munich, Germany; 2 Department of Dermatology and Allergy, Hannover Medical School, Hannover, Germany; 3 Division of Dermatology and Venereology, Department of Medicine Solna, Karolinska Institutet, Stockholm, Sweden

**Keywords:** Atopic dermatitis, Consumer behavior, Skin care, Social media, Qualitative research, Public health

## Abstract

**Background:**

Atopic dermatitis (AD) is a chronic inflammatory disease with a complex pathophysiology, necessitating strict therapeutic management. Over-the-counter products play a crucial role in AD treatment. The emergence of social media (SM) as a vast information source for skincare and healthy lifestyle has transformed its role from mere entertainment. This study aimed to explore the attitudes of AD patients towards SM as an information source for their AD products, understand their purchase behavior, and identify unmet needs.

**Methods:**

Qualitative semi-structured interviews were conducted with AD patients recruited from a university hospital in Southern Germany and social media networks. The interviews took place between November 2022 and January 2023. The recorded interviews were transcribed verbatim and analyzed using Mayring’s qualitative content analysis.

**Results:**

A total of ten patients (3 men, 7 women) aged 23–42 years were interviewed. Participants reported new perspectives in four categories: opportunities and advantages of SM as information source for AD products, risks and disadvantages, important aspects for patients’ choice of AD products, and extent and purpose of SM use in selecting AD skincare.

**Conclusions:**

Patients exhibit diverse patterns of SM use when selecting daily products and critically evaluate the online content, demonstrating a greater trust in healthcare professionals or familial connections. Electronic word-of-mouth, disease severity and prior product experiences emerge as prevalent factors influencing patients’ product selection. Furthermore, female patients express interest in complementary and alternative therapies as part of an integrative therapeutical approach. Understanding patients’ needs and preferences regarding AD skincare can inform physicians in recommending more personalized therapies. Additionally, educational interventions on SM, addressing patients’ questions and concerns with evidence-based information, hold the potential for beneficial outcomes.

**Supplementary Information:**

The online version contains supplementary material available at 10.1186/s12889-025-21640-8.

## Background

Atopic dermatitis (AD, syn. atopic eczema) is one of the most common skin diseases, showing a prevalence of 2-10% in adults and up to 20% in children [1].

As a chronic inflammatory skin disease, AD requires complex therapy with short-term control of flares and long-term stabilization of the overall skin quality [2]. European guidelines propose a 4-step therapy plan, with a basic therapy consisting of educational programs, emollients, bath oils, and avoidance of clinically relevant allergens, if diagnosed by allergy tests [3]. It was shown that topical emollients have positive effects on the overall skin quality such as reduction of transepidermal water loss and xerosis cutis [4], a positive influence on the disease exacerbations [5], and a decrease in usage of topical glucocorticoids [6]. Underlining the importance of baseline therapy in the ADcare, it is important to address the challenges the patients might encounter while selecting the appropriate products, such as having to choose from a vast product variety available on the market or interpreting the list of ingredients by themselves [7]. Furthermore, 40-65% of AD patients might suffer from a contact allergy [2]; metals, fragrances, and emollients are among the most common contact allergens and higher rates of contact sensitizations correlate with higher disease severity scores [8]. Therefore, a constant basic therapy, adapted to the skin condition and with a low allergenic potential is recommended [9].

Social media (SM) platforms have become increasingly influential in recent years, with 4.8 billion registered SM users worldwide as of April 2023 [10]. As defined by Kaplan and Haenlein, SM includes “a group of Internet-based applications that build on the ideological and technological foundations of Web 2.0, and that allow the creation and exchange of User Generated Content” [11]. Within the community perspective, online social networks can be regarded as communities of individuals with common interests, serving not only social purposes but also economic and commercial ones. As a result, e-commerce sites evolve as virtual commercial communities, blurring the boundaries between SM and e-commerce [12].

Besides changing the way we interact, SM also transformed how medical information becomes available to us, contributing to a “Democratization of Health Care” [13] and inverting the patient-physician relationship [14]. This aspect of SM use in healthcare was approached in several studies in the past years. The use of these alternative sources does not impact the physician-patient relationship in a negative way, but is more a complementary means of information [15, 16], a positive health mediator if its negative effects are minimized [17].

Other studies analyzed what AD patients mostly search for in the online environment and in which aspects they demand a wider education [18, 19], revealing that quality of life, disease and financial burden, as well as recommendations for AD management, were among the most discussed topics in the online media [19]. A cross-sectional analysis of videos about AD on the SM platform TikTok reported that 45.5% of the posts made a treatment or product recommendation, a third of them being made by patients themselves, followed by skincare companies, board-certified dermatologists and influencers [20]. Furthermore, SM also facilitates monitoring the public disease burden in prevalent diseases such as allergic rhinitis, with SM post volume as a correlate for disease burden [21].

Some patients show mistrust in physicians and evidence-based therapies such as corticosteroids [20], an attitude that might be also fueled by a range of misleading information in SM [22]. While experts clearly formulate a general framework and guidelines for accurate therapies and specific products for AD [2], it was not thoroughly researched if and to which extent patients are aware of these evidence-based therapies. The impact of SM on patients’ preferences for AD products emerges therefore as an aspect that requires further investigation.

In this study, we explored AD patients’ experiences and attitudes toward SM in the context of the decision-making process for selecting specific AD skincare products.

## Methods

### Study design

The established standards for reporting qualitative research (i.e., SRQR-Standards for Reporting Qualitative Research) [23] were followed in this study. In-depth, semi-structured interviews among individuals with AD were conducted between November 2022 and January 2023 in Germany. The inclusion criteria were self-reported physician-diagnosed AD, age ≥ 18 years and any SM use. Language barriers (unable to conduct a German interview) were the exclusion criterion. Since the participants’ locations were spread within several regions in Germany, the interviews were conducted either face-to-face or online.

### Ethical review

The study was reviewed and approved by the ethics committee of the School of Medicine of the Technical University of Munich (reference 2022-460-S-SR) and in accordance with the Declaration of Helsinki. Written informed consent was obtained from all the participants prior to inclusion. The participants were informed about the purpose of the study, the use of their data, their rights as participants, the researcher’s characteristics (name, position, and research interest), and had the possibility to ask questions. The interviewer was not familiar with the participants prior to the study. There were no incentives offered for participation in the study.

### Recruitment and interview group

The participants were recruited at the Department of Dermatology and Allergy at the Technical University of Munich, as well as via online SM channels (AD networks, self-support groups, influencers). Potential participants were either approached by the interviewer (R.M. female, doctoral candidate, trained in qualitative research) at the clinic or they contacted the interviewer themselves at the email address provided in the information flyer posted online.

Purposive sampling was used to create a diverse population regarding gender, age, and disease severity. The latter was registered as patients’ subjective assessment with the options “mild”, “moderate” and “severe”. Recruitment ceased when data saturation was attainted, i.e., data redundancy [24] was identified in the interview texts and no further codes within the categories were emerging from the data.

### Data collection

Face-to-face interviews with stationary patients took part in a quiet enclosed room in the clinic, while the online interviews were conducted via Zoom (Video Communications, Inc. San Jose, CA, USA), in both cases without anyone else being present. The interviews were conducted in German and no interview hat to be repeated.

All interviews were audio-recorded. To encourage a connection between participant and interviewer, the online meetings were held with a turned-on camera, however, only the audio files were used for data analysis. The interviews ranged between 30 and 60 min in length. Field notes were taken by the interviewer after concluding each interview.

Using relevant literature and the manual for conductive qualitative interviews by Helfferich [25], a topic guide including open-ended questions was generated in order to cover themes identified as important for the research question (**Additional** Table 1). Although not typically seen as “social media”, we included e-commerce platforms in this context, as they can be regarded as virtual communities where users interact through reviews and testimonials.

**Table 1:** Patients’ characteristics

Patient number	Gender	Age (years)	Place of living	AD-severity ^a^	Recruitment setting	SM use for AD
P01	female	30	metropolitan area	severe	online	yes
P02	male	32	towns/suburb area	severe	inpatient	no
P03	female	34	metropolitan area	mild	online	yes
P04	female	23	towns/suburb area	mild	outpatient	yes
P05	male	35	metropolitan area	moderate	inpatient	only e-commerce ^b^
P06	male	42	metropolitan area	mild	outpatient	only e-commerce ^b^
P07	female	30	metropolitan area	severe	outpatient	yes
P08	female	33	metropolitan area	moderate	online	only e-commerce ^b^
P09	female	23	regional/rural	moderate	outpatient	yes
P10	female	29	metropolitan area	moderate	online	yes

Abbreviations: AD, atopic dermatitis; SM, social media; ^a^, registered as patients’ subjective assessment; ^b^, patients reporting using solely e-commerce platforms (e.g., online pharmacies)

### Data processing and analysis

Audio-recorded interviews were transcribed verbatim by R.M. following transcription rules in accordance with Kuckartz [26]. All transcripts were anonymized, receiving an ID number.

The interview data was analyzed according to Mayring’s qualitative content analysis [27] to maintain the explorative character of the research. The first themes emerged through familiarization with the complete set of transcripts. A preliminary coding scheme was created, using the interview guide for the deductively generated topics and line-by-line analysis of the text material for inductively generated topics. A further development of the coding grid was achieved through paraphrasing and systematic reduction. Redundancies in the coding scheme and alternative interpretations of the codes were discussed among R.M. and S.Z. until reaching a general agreement.

In order to enhance the inter-coder ratability, two interviews (20%) were coded individually by R.M. and S.Z. The calculated Cohen’s Kappa of 0.85 was considered adequate [27]. The authors revised and discussed the uncertainties (R.M., S.Z.) No further adjustments were made to the coding scheme and all the remaining transcripts were coded by R.M.

Transcription, data management, and analysis were performed using the qualitative data analysis software MAXQDA 2022 (VERBI Software, Berlin, Germany). The most representative quotes selected for reflecting the findings were translated from German to English and included in the presentation of results.

## Results

Ten persons with AD participated (7 women, aged 23–42 years, 40% recruited via SM; Table 1). Most of the patients admitted having used SM as an information source for AD or AD-related products. The degree of involvement varies from none, solely use of e-commerce platforms to regular information-seeking behavior regarding AD.

**Fig. 1:**
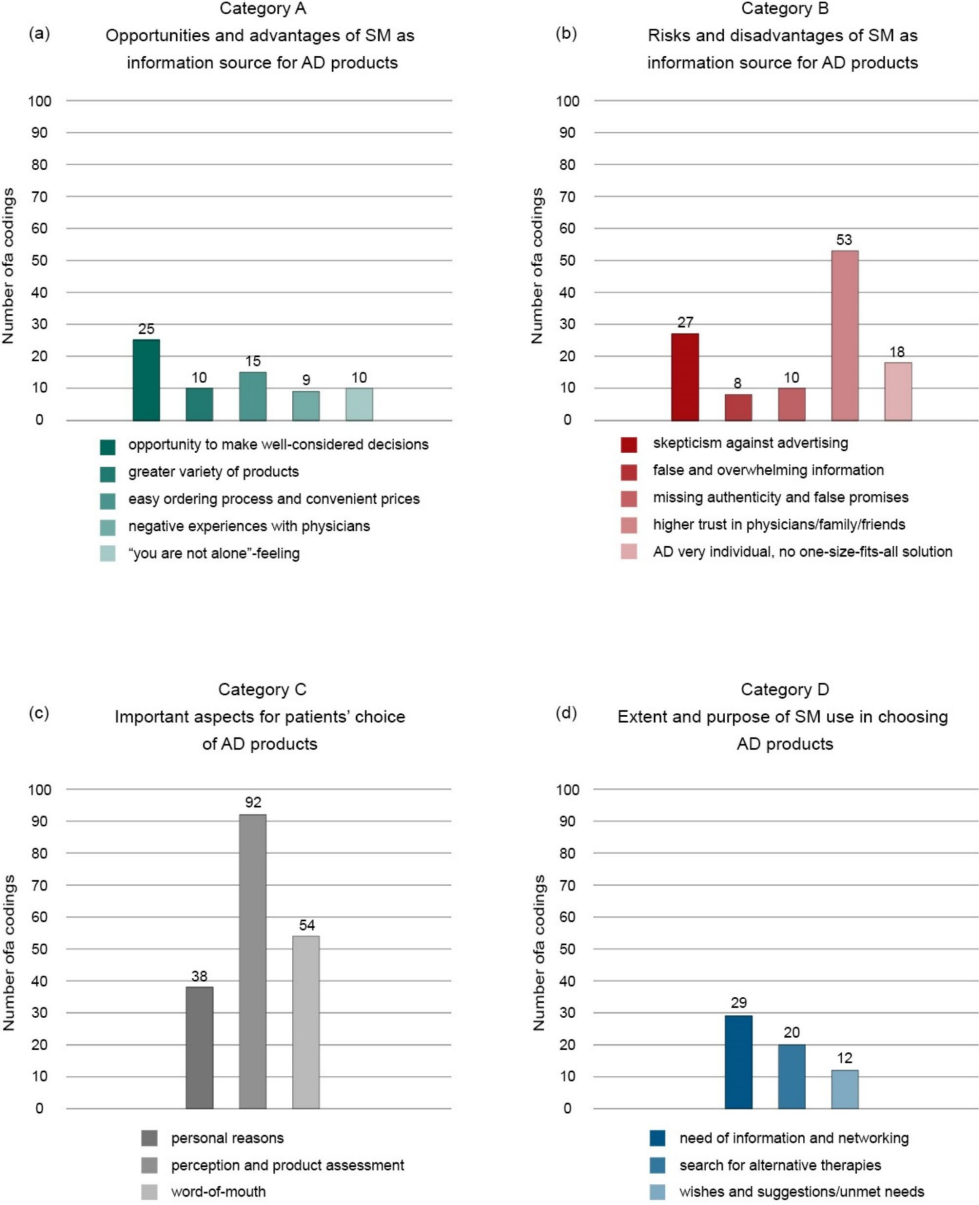
Frequency of different codes within categories. (**a**) Category A - Opportunities and advantages of SM as information source for AD products, (**b**) Category B - Risks and disadvantages of SM as information source for AD products, (**c**) Category C - Important aspects for patients’ choice of AD products, (**d**) Category D - Extent and purpose of SM use in choosing AD products. Abbreviations: AD, atopic dermatitis; SM, social media

Four main categories emerged: (a) opportunities and advantages of SM as information source for AD products, (b) risks and disadvantages of SM as information source for AD products, (c) important aspects for patients’ choice of AD products, and (d) extent and purpose of SM use in choosing AD products (Fig. 1).

### Opportunities and advantages of SM as information source for AD products

The majority of the participants reported seeing in SM the opportunity to make thoughtful decisions, including a comprehensive search for information prior to product purchase, suitability for one’s own needs, time independence, and review of products recommended by physicians, friends, or family. Other advantages of SM regarding the choice of AD products were seen in the great variety of products, with some niche products (e.g., clothing for AD) only being available online, an easy online ordering process, and convenient prices.

Three female participants mentioned that negative experiences with physicians or a lack of individualized therapy from their physician were the reasons for turning to SM for choosing products and alternative therapies.

*“I think that you don’t just have to use creams for neurodermatitis*,* you also have to work a bit on your own mental attitude*,* on your lifestyle*,* and I would say I learned more from social media than from the doctors.” (P10)*.

Participants seemed to appreciate SM as a space generating inclusion and emotional support, where self-affected SM players are perceived as trustworthy through their own experiences, promoting self-confidence and acceptance of the disease among users.

### Risks and disadvantages of SM as information source for AD products and their consequences

All participants expressed skepticism about advertising, the potential profit orientation of SM, and the reliability of information on SM and mentioned that enhanced transparency would make SM a more adequate source for medical information. Most of the patients recruited in the clinic, favor reputable sources (e.g., known newspapers, official university websites) to typical SM platforms.

An overwhelming and sometimes dangerous information was emphasized by some participants, who declared that the flood of opinions and products in SM make the decision for a product difficult and led to losing track of therapy.

*“(…) And also*,* a simply too large range of things*,* products and a great flood of opinions (…)*,* and it is sometimes difficult to decide for yourself what to choose or which product to buy.” (P07)*.

Patients appreciated the individual counseling in the offline setting (i.e., on-site pharmacies) mentioning that a one-size-fits-all solution for all AD patients, as promoted in SM, seems unrealistic and almost all respondents consequently stated a higher trust in physicians, pharmacists, family, and friends compared to SM.

### Important aspects for patients’ choice of AD products

Most participants confessed low product efficacy and high suffering as a driver to purchase new products.

*“Unplanned purchases… (…) my skin would have to feel extremely bad (…) and then my level of suffering would have to be greater or maybe a certain frustration that something else didn’t help.” (P03)*.

Good quality perception indicated as natural ingredients, significant product description, medical skincare, atopic skin labeling, promising price-performance ratio, authentic advertising, and a positive experience with certain products and brands were discussed as indicators for the purchase decision. Electronic word-of-mouth was emphasized as an essential aspect of the decision-making process when buying AD products, to assess possible side effects, poor product quality, or difficult ordering process, even for products recommended by the physician or pharmacist. Nevertheless, some participants were critical of reviews and testimonials for various reasons: faked customer reviews and limited significance due to the high variability in the effectiveness of skincare products.

### Extent and purpose of SM use in choosing AD products

Half of the participants describe using SM in need of information and networking. The sought information in SM ranges from existing therapies and products for AD, possible causes, research developments in the field, to cost deduction opportunities. Alternative therapies were also mentioned, female patients showing interest in AD apps, special clothing, complementary products, or special diets.

Some patients mentioned the need for a single digital platform with evidence-based information in layman’s language, more active physicians in the SM promoting educative material, promotion of body-positivity, disease awareness, and higher public attention on how patients use SM for their AD-related information. Patients also mentioned they wish for a more holistic approach to AD therapy from their physicians, with counseling on nutrition and a healthy lifestyle adjoining the drug therapy.

## Discussion

The present comprehensive qualitative investigation elucidates the experiences and attitudes of individuals with AD towards SM within the intricate process of decision-making regarding the acquisition of AD skincare products. Notably, the study discerns the significance of electronic word-of-mouth, encompassing product reviews, and testimonials, as a pivotal factor influencing the selection of skincare items, notwithstanding the patients’ inclination to place greater trust in healthcare providers or familial connections. While SM affords patients the opportunity to access well-founded insights for informed decision-making, a certain degree of apprehension persists regarding the reliability of AD-related information disseminated through these platforms. Furthermore, the investigation underscores additional crucial considerations for AD product selection, encompassing disease severity, previous experiential factors, optimal quality, and a favorable price-performance ratio. In addition, female patients exhibit a notable interest in embracing complementary and alternative therapies as an integral facet of their holistic approach toward skincare.

The extent to which patients use SM to inform themselves about their AD products seemed to depend primarily on the perceived opportunities and risks, as well as on their trust in the SM as a source of medical information. Electronic word-of-mouth in the form of customers’ reviews and testimonials introduces quality, credibility, and authenticity in SM [28]. Our results show that AD patients consider product reviews when selecting skincare in order to reduce their perceived risk, confirming that users’ reviews play together with price a defining role in their willingness-to-pay [29]. As several studies proved that electronic word-of-mouth has an impact on the users’ purchase intention [30], further research on how word-of-mouth might influence AD patients’ purchase behavior could be beneficial in understanding the impact of SM on patients’ financial burden.

Nevertheless, SM should not be seen as a way for patients to bypass physicians, but a complementary source of health-related information [31]. In accordance with previous findings [15], trust in physicians, family, or friends, when choosing AD skincare, emerged as a unanimous statement of the present study. Patients confirmed that untrustworthy, potentially dangerous information in SM is a reason for the lack of trust in SM content on skincare, even though they are aware of online marketing strategies. Other studies revealed patients’ mistrust in dermatologists and evidence-based therapies [20], as well as several common conspiracy theories and misleading information on AD in the online media [32].

The important aspects identified in our study for patients’ choice of AD products are consistent with previous findings on moisturizers, which vary widely in terms of price, allergenicity, and patients’ preferences, showing patients’ concern for qualitative ingredients [7]. Since not all natural ingredients are beneficial to the atopic skin, some being suspected of affecting the already damaged skin barrier [33], patients should therefore receive professional advice from their physicians to select emollients with few ingredients, hypoallergenic and suitable to their own preferences and needs, regarding age, affected body area, acuteness, and climate [34].

Another important aspect of AD management is the patients’ financial sacrifice, since moisturizers and emollients for adults are not covered by the health insurance companies in Germany [9]. Studies reported a significant willingness-to-pay in AD patients [35] and a higher willingness-to-pay correlating with a more impaired health-related quality of life [36]. These results are sustained by our study, which suggests that disease severity emerges as a driving factor influencing patients’ product selection. This is an important issue to take into consideration in attempting to minimize the out-of-pocket costs of AD patients and a widely effective dermatological care for all AD patients should be strived for.

### Strengths and limitations

While in-depth interviews provide detailed data and insights on patients’ position on the topic, the small sample as frequently found in qualitative studies does not allow a generalization of the findings. The achieved sample was heterogeneous in disease severity, however not in terms of sex, age, and education. This self-selection bias could be explained by the participants’ interest in the study topic and by women being more likely to search for health-related information in the online media than men [37]. Nevertheless, data saturation was reached and patients from different healthcare settings were included. Information bias is not to be ruled out, since participants mainly associate SM with the most well-known platforms (e.g., Facebook, Instagram) disregarding other sources (e.g., blogs, forums). Furthermore, it was not always clear whether the use of SM for AD products referred to purchasing or only information-seeking behavior. The recruitment process was conducted through both offline methods - at the Department of Dermatology and Allergy at the Technical University of Munich - and online platforms - utilizing SM channels. Notably the online recruiting strategy was regarded as highly adequate for this study, facilitating engagement with a very specific group - AD patients using SM. As previously discussed in the literature, this recruiting approach is particularly effective for observational studies, hard-to-reach or very specific target populations [38].

## Conclusions

In conclusion, our study adds new insights into the AD patients’ purchase behavior and the impact of SM in their decision-making process. Patients used SM very differently in choosing their daily AD products, but all patients seemed to interpret the online content critically. Nevertheless, physicians should understand the risks and opportunities of SM as a medical information source and address their patients’ questions and concerns about the online information on AD. Furthermore, a better understanding of patients’ preferences for AD products, might help physicians empower their patients and make the right decisions regarding basic therapy. We also postulate that a more integrative therapy approach, including recommendations on healthy lifestyle, dietary measures, alternative therapies, and educational SM platforms with evidence-based information on AD could be beneficial for patients.

## Electronic supplementary material

Below is the link to the electronic supplementary material.

**Supplementary Material 1**: **Additional Table 1**-Interview topic guide, **Additional Table 2**-Final coding system.

## Data Availability

Interview transcripts are available from the authors upon reasonable request. Analysed data is provided within the manuscript or supplementary information files.

## Abbreviations

AD: Atopic dermatitis
SM: Social media
