# Supplementary material for: Patients’ preferences on atopic dermatitis skincare and social media use: a qualitative study
Source: BMC Public Health. 2025 Feb 5;25:467. doi: 10.1186/s12889-025-21640-8 (PMC11800506; doi:10.1186/s12889-025-21640-8)
Supplement: Supplementary file 1 — Supplementary Material 1: Additional Table 1-Interview topic guide, Additional Table 2-Final coding system. [file 12889_2025_21640_MOESM1_ESM.docx]

## Additional Table 1. Interview topic guide

| **Main questions** | **Detailed questions** | **Aim** |
| --- | --- | --- |
| *A. Open question:* | | |
| Describe your personal experience with SM as AD patient, relating to you condition. | Please mention which SM platforms did you use, how did you use them and which role did they play in your purchase decisions for AD products. | Understanding the patients’ experiences in context of SM use for their AD products and their perspective to the topic. |
| *B. WHERE* | | |
| Where do you buy your AD products? | How much of your total purchases related to AD occur online?  Depending on previous answer: why do you prefer buying your AD products online/offline? | Identifying the preferences -online vs. offline purchases- and the reasons for/against them. |
| Where do you get your information about AD products? | On which platforms do you get your information about your AD products?  Describe the purchase process you follow when buying your AD products. | Identifying the SM platforms, where the patients get their information on AD products and how the purchase of these products is conducted. |
| *C. WHY* | | |
| Which SM features have a role while searching for your AD products online? | Please describe what is especially important to you while searching for AD products through SM. | Understanding which SM features are important for the patient when choosing an AD product. |
|  | Please describe the meaning of the word-of-mouth in SM for your purchase decisions of AD products. |  |
| What makes you pay more for an AD product? |  |  |
| How would you describe your purchase behavior related to new AD products or brands? | Please describe the determining factors that play a role in your decision to try new AD products/ change your current product. | Understanding how eager patients are to try out new AD products and which are the reasons that make them open to new products/brands. |
| What makes you make unplanned purchases while buying your AD products online? | Which factors are more important than price when choosing your AD products through SM? |  |
| *D.WHAT* | | |
| Which product categories for AD do you mostly buy through SM? |  | Identifying which categories of AD products are mostly bought through SM. |
| *E. PATIENT PERSPECTIVE* | | |
| What is your general point of view on SM regarding the purchase of you AD products? | What do you like the most/the least in SM as an information source for your AD products? | Understanding the patients’ attitudes towards SM as an information source for their AD products. |
| How likely is that you will inform yourself and buy your AD products through SM in the future? | Taking your previous experience with SM in buying your AD products into account, how would you evaluate the influence of SM in your purchase decision? | Identifying the purchase intention of AD products through SM in the future. |
| Do you have additional comments or wishes on the topic? |  | Identifying patients’ unmet needs regarding SM as an information source for AD and potential improvements in this regard. |
| *F. SOCIAL MEDIA LITERACY* | | |
| How much time do you spend weekly on SM?  How long have you been using SM?  How would you describe your SM literacy? | Please describe your competency in managing your SM accounts, evaluate, share and create SM content, critical information analysis, awareness of privacy policies and SM algorithms. | Identifying relevant factors that describe the extent of patients’ SM use. |
| *G. SOCIAL MEDIA ENGAGEMENT* | | |
| How do you interact with other users in SM regarding your AD? | Please describe which SM tools do you use when interacting in SM (likes, shares, posts) and to which extent. | Identifying relevant factors that describe the pattern of patients’ SM use. |
| *H. DEMOGRAPHICS* | | |
| Gender  Age  Place of living  Highest level of education  Time of diagnosis  Self-reported severity level |  |  |

AD, atopic dermatitis; SM, social media

## Additional Table 2. Final coding system

| **Categories** | **Nr. of codings** |
| --- | --- |
| **A. Opportunities and advantages of SM as information source for AD products** | **69** |
| - opportunity to make well-considered decisions - greater variety of products - easy ordering process and convenient prices - negative experiences with physicians - “you are not alone”-feeling | 25  10  15  9  10 |
| **B. Risks and disadvantages of SM as information source for AD products** | **132** |
| - skepticism against advertising, trustworthy media preferred - false and in part dangerous information, an overwhelming amount of information to sort out - missing authenticity and false promises - more trust in physicians/family/friends - AD very individual, no one-size-fits-all solution, individual counselling offline | 43  8  10  53  18 |
| **C. Important aspects patients’ choice of AD products** | **184** |
| - **Personal reasons** - eagerness/having a good day - loss of effectiveness of previous AD-products - poor skin condition/acute flare-up/impairment in quality of life - **Perception and product assessment** - perceived quality: promising, good/natural ingredients, advertising authenticity - previous experience with the product/brand - monetary sacrifice   **Word-of-mouth** | **38**  11  11  16  **92**  61  14  17  **54** |
| **D. Extent and purpose of SM-use in context of choosing AD products** | **61** |
| - need of information and networking - search for alternative therapies - wishes and suggestions/unmet needs | 29  20  12 |

AD, atopic dermatitis; SM, social media
